# Styrene–Acrylic Elastomeric Waterproofing Membranes: Composition, Performance, Durability and Emerging Formulation Technologies

**DOI:** 10.3390/polym18111390

**Published:** 2026-06-03

**Authors:** Artemis Kontiza, Maria Pastrafidou, Ioannis A. Kartsonakis

**Affiliations:** 1 Department of Mechanics, School of Applied Mathematical and Physical Sciences, National Technical University of Athens, 15780 Athens, Greece; kontiza.artemis@gr.sika.com; 2 Sika Hellas ABEE, Protomagias 15, 14568 Kryoneri, Greece; 3 Laboratory of Physical Chemistry, School of Chemistry, Aristotle University of Thessaloniki, 54124 Thessaloniki, Greece; mpastraa@chem.auth.gr

**Keywords:** water-based elastomeric membranes, styrene–acrylic copolymers, liquid-applied waterproofing, crack-bridging performance, UV and weather resistance, VOC reduction/eco-friendly coatings, acrylic–styrene blends, polymer dispersion technology, impermeability enhancement, durability in waterproofing systems

## Abstract

Water-based elastomeric waterproofing membranes based on styrene–acrylic (S/A) copolymers have emerged as an important class of materials for modern construction due to their combination of flexibility, adhesion, environmental compatibility, and long-term durability. These membranes form seamless protective layers capable of accommodating substrate movement while preventing water ingress across a wide range of building structures. Recent advances in polymer chemistry and emulsion technology have significantly improved the performance of S/A systems, particularly in terms of crack-bridging capability, weather resistance, and UV stability. In addition, optimized formulations incorporating functional fillers, rheology modifiers, and hybrid polymer architectures enable improved mechanical performance and impermeability. This review provides a comprehensive overview of S/A elastomeric waterproofing membranes, covering polymer chemistry, formulation strategies, physico-mechanical properties, durability mechanisms, and real-world construction applications. The review also compares S/A systems with alternative waterproofing technologies such as polyurethane (PU), cementitious coatings, and bituminous membranes. Finally, emerging developments in advanced polymer architectures, nano-reinforced coatings, and sustainable formulations are discussed, highlighting future research directions for high-performance waterproofing systems.

## 1. Introduction

### 1.1. Liquid-Applied Waterproofing Membranes

Liquid-applied waterproofing membranes (LAMs) are fluid coatings, typically polymer-based, that are applied cold and form a seamless, fully bonded, monolithic waterproof barrier upon curing. Compared with sheet membranes [bituminous, polyvinyl chloride (PVC), thermoplastic polyolefin (TPO), ethylene propylene diene monomer (EPDM)], LAMs eliminate overlaps and weld failures while providing excellent detailing capacity for complex geometries. They can be applied to concrete, masonry, wood, metal, and composite substrates, and their flexibility makes them suitable for both new construction and refurbishment projects [1,2,3].

LAM technologies include polymer-modified cementitious membranes, acrylic and styrene–acrylic (S/A) membranes, styrene–butadiene (SBR) emulsions, PU and polyurea systems, hybrid formulations, and epoxy-based coatings. Each category offers a distinct balance between elongation, tensile strength, water impermeability, and resistance to ultraviolet (UV) exposure. Water-based acrylic and S/A systems are particularly prevalent due to their strong wet adhesion, flexibility, crack-bridging behavior, and compliance with key waterproofing standards such as DIN EN 14891 and ETAG 022. PU-based liquid membranes also remain widely used, especially in exposed high-wear applications such as balconies, podium decks, and pedestrian surfaces, owing to their proven 20-year performance record.

LAMs are increasingly favored in the construction industry due to their ready-to-use, solvent-free, and low-VOC formulations, as well as ease of application and compliance with modern waterproofing standards (EN 14891, ETAG 022). Industrial application methods such as roller, brush, and airless spray play a critical role in determining the final performance of S/A waterproofing membranes. These techniques impose different shear histories and deposition conditions, which influence dispersion rheology during application and subsequent film formation. Roller and brush applications generally provide higher control over coating thickness but may introduce defects such as air entrapment or uneven coverage, particularly on rough substrates. In contrast, airless spray enables rapid and uniform application over extensive areas, although improper atomization or spray parameters can lead to over-spray, pinhole formation, or thickness variability. Film thickness uniformity is a key parameter affecting barrier properties and mechanical performance, while defects such as voids or discontinuities can act as pathways for water ingress. Additionally, curing behavior is influenced by application thickness and environmental conditions (temperature, humidity), which govern water evaporation rate and polymer particle coalescence. Therefore, optimization of application methods and parameters is essential to ensure consistent film formation and long-term durability.

Volatile organic compounds (VOCs) are organic chemical species that evaporate readily under normal conditions due to their relatively high vapor pressures and low boiling points and are commonly produced by solvents and other formulation components in polymer coatings and waterproofing systems. The specific VOC content and emission profile of a styrene–acrylate waterproofing composition depend on the exact combination of monomers, solvents, and additives used, with water-based and solvent-based systems exhibiting significantly different VOC characteristics and emission factors [4,5]. Compared to sheet-type membranes (bituminous, PVC, TPO, EPDM), LAMs offer enhanced crack-bridging capacity, superior adhesion, and versatile applicability across roofs, facades, wet rooms, foundations, and retaining walls [6,7]. Recent studies indicate that while sheet membranes provide high mechanical stability, LAMs achieve comparable durability and water resistance while allowing simpler detailing and faster installation, making them competitive alternatives in both new construction and refurbishment projects [8].

### 1.2. Importance of Water-Based S/A Elastomeric Systems in Modern Construction

Water-based S/A elastomeric dispersions have become a cornerstone technology for waterproofing, largely due to their combination of flexibility, crack-bridging, UV resistance, low water uptake, and excellent adhesion. Their film-forming behavior enables the creation of robust elastomeric layers that maintain integrity under substrate movement and thermal cycling [6,9].

Industry leaders highlight the performance advantages of S/A emulsions. When S/A dispersions are applied onto aged substrates, a combination of mechanical interlocking, physicochemical interactions, and limited interdiffusion at the interface governs adhesion. Aging processes such as UV exposure, oxidation, plasticizer loss, and surface contamination can significantly alter surface energy and reduce the availability of active functional groups, thereby impairing wetting and adhesion. In previously applied S/A films, partial compatibility may still allow interpenetration of polymer chains; however, this is often restricted by prior crosslinking or embrittlement. For dissimilar polymeric substrates, adhesion depends strongly on polarity matching and interfacial tension. Proper surface preparation is therefore critical and typically includes cleaning to remove contaminants, mechanical roughening to enhance anchorage, and, where necessary, the use of primers to restore surface reactivity and improve interfacial bonding. Failure to adequately prepare aged surfaces can result in interfacial defects, reduced cohesion, and premature delamination under service conditions.

Synthomer’s SA dispersions, for example, are described as solvent-free, low-odor, low-VOC, and capable of delivering superior water resistance, tensile strength, flexibility, and excellent wet adhesion while meeting ETAG 022 and DIN EN 14891 quality standards. Likewise, Dow identified S/A and pure acrylic emulsions as central to high-performance ready-to-use waterproofing systems, providing durability, crack-bridging, and excellent adhesion to concrete in wet rooms, balconies, and water-retaining structures. BASF also emphasized acrylic latex polymers as key components in waterproofing membranes, enhancing strength, elongation, flexibility, moisture vapor permeability, and adhesion requirements for reliable building envelope protection [7].

Water-based S/A elastomeric systems are increasingly preferred for both internal wet areas and exterior waterproofing applications due to their sustainability, ease of application, and long-term durability. Comparative evaluations indicate that S/A emulsions provide high elongation, strong adhesion, and weather resistance, performing on par with or better than traditional binder systems. Their versatility across wet rooms, balconies, water tanks, and facades supports widespread adoption in both new construction and refurbishment projects [10,11].

Because they are water borne, these systems align with industry demands for safer application, lower emissions, easy cleanup, and reduced fire/health risks compared with solvent-borne counterparts. Their versatility allows use in: internal wet areas (bathrooms, kitchens, laundry rooms), balconies and terraces, roofs (flat/sloped), facades and exterior walls, pre-tiling substrates, and decorative/functional protective coatings. The flexibility and environmentally compliant profile of S/A waterproofing membranes have made them default materials in many countries with strict VOC and sustainability standards.

### 1.3. Environmental, Regulatory, and Performance Drivers (VOC Limits, Sustainability, Longevity)

Stricter air-quality and occupational health regulations worldwide have accelerated the transition from solvent-borne waterproofing systems toward low-VOC, water-based technologies. Many S/A waterproofing membranes are formulated to be solvent-free, free of film-forming aids, and compliant with low-VOC thresholds under modern regulatory frameworks. This aligns not only with EU and US regulatory standards but also with voluntary sustainability certifications such as Leadership in Energy and Environmental Design (LEED), Building Research Establishment Environmental Assessment Method (BREEAM), and WELL Building Standard [12].

Sustainability criteria increasingly influence design choices in construction. Acrylic-based LAM systems are viewed as environmentally preferable due to their reduced toxicity, non-flammability, low embodied energy, and compatibility with “green building” specifications. Manufacturers such as BASF and Dow emphasize their acrylic dispersions as durable, long-lasting, and weather-resistant, enabling extended lifecycle performance of waterproofing assemblies while reducing long-term material consumption and waste generation.

Beyond environmental benefits, water-based S/A membranes are chosen for their robust performance characteristics, including excellent crack-bridging and flexibility, wet adhesion to concrete and cementitious substrates, UV and weathering resistance, water impermeability even under hydrostatic pressure, and compatibility with tile adhesives and cementitious mortars. Such properties make S/A systems suitable for both interior and exterior applications, and compliant with the increasing technical demands for long-term durability and reliability of building envelopes.

### 1.4. Objectives and Structure of the Review

**Figure 1:**
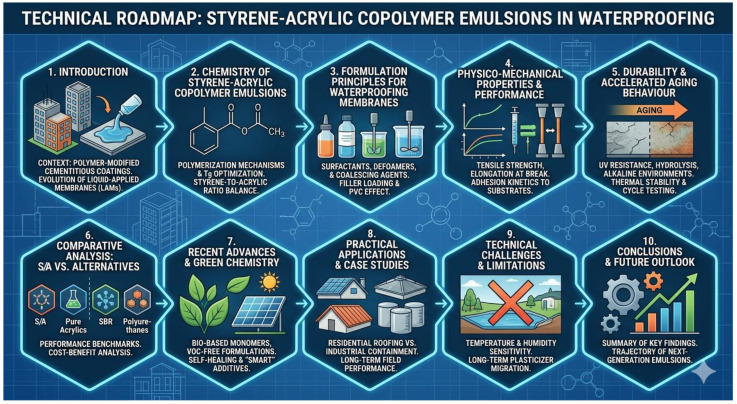
The framework of this review article.

The present review aims to provide a comprehensive examination of liquid-applied waterproofing membranes, with a dedicated focus on water-based styrene–acrylic elastomeric systems (Figure 1). The primary objectives are:

1. To summarize current LAM technologies, their formulations, and their performance characteristics across major market segments.
2. To analyze environmental and regulatory factors, including VOC restrictions, sustainability imperatives, and durability expectations that shape the development of modern waterproofing systems.
3. To evaluate the performance of S/A membranes, examining crack-bridging, adhesion, waterproofing behavior, weatherability, and comparative advantages relative to PU, polyurea, cementitious, and hybrid systems.
4. To discuss formulation principles, including polymer selection, additives, fillers, and rheological design, that influence film formation and membrane performance.
5. To review industrial data and commercial technologies, referencing performance attributes of products from Synthomer, BASF, Dow, Sika, and other industry players.
6. To identify research gaps and future opportunities, particularly in low-VOC material innovations, enhanced durability systems, multifunctional coatings (e.g., UV-reflective, antimicrobial), and advanced characterization techniques.

## 2. Chemistry of S/A Copolymer Emulsions

Among the most widely used binder systems for water-based elastomeric waterproofing membranes are S/A copolymer emulsions due to their favorable combination of flexibility, adhesion, weather resistance, and environmental compatibility. These polymers are typically synthesized through emulsion polymerization, a heterogeneous free-radical polymerization process that enables the formation of stable polymer particles dispersed in water. The resulting latex dispersions provide excellent film-forming capability after water evaporation, allowing the formation of continuous and elastic protective membranes on mineral and metallic substrates.

Compared with solvent-borne polymer systems, water-based S/A dispersions offer several environmental and technological advantages, including reduced volatile organic compound (VOC) emissions, improved safety during application, and compatibility with modern sustainable construction practices. As a result, they have gained widespread use in coating applications, adhesives, and waterproofing systems used in building envelopes and infrastructure protection [13,14,15].

The final properties of S/A waterproofing membranes depend strongly on polymer chemistry, including monomer composition, particle morphology, glass transition temperature (T_g_), and the presence of stabilizers and additives. Through careful control of these parameters, polymer scientists can tailor materials with optimized elasticity, mechanical strength, and durability for demanding construction applications.

### 2.1. Fundamentals of Emulsion Polymerization

Emulsion polymerization is one of the most important industrial processes for producing polymer latexes used in water-borne coatings and construction materials. The process involves the polymerization of hydrophobic monomers in an aqueous medium using surfactants and water-soluble initiators such as persulfates [16]. The mechanism typically proceeds through three major stages: nucleation, particle growth, and stabilization. Initially, surfactants form micelles in the aqueous phase, which serve as nucleation sites for polymer particles. Free radicals generated by the initiator diffuse into these micelles and initiate polymerization of the monomer molecules. As polymerization proceeds, monomers diffuse from the aqueous phase into the growing particles, resulting in the formation of stable polymer latex particles typically ranging from 50 to 300 nm in diameter [17].

The resulting latex dispersions exhibit excellent stability and film-forming behavior after water evaporation. During film formation, the polymer particles undergo deformation and coalescence, producing continuous elastomeric film capable of providing waterproofing protection and mechanical flexibility. Compared with other polymerization techniques, emulsion polymerization offers several advantages, including high-molecular-weight polymers, efficient heat transfer, and environmentally friendly water-based processing. These characteristics have made the technique the dominant manufacturing route for S/A dispersions used in waterproofing membranes and architectural coatings [18].

### 2.2. Role of Monomer Selection (Styrene, Acrylics, Crosslinkers)

The performance of S/A copolymers is primarily determined by the selection and ratio of monomers used during polymerization. Styrene is commonly incorporated into acrylic copolymers to increase mechanical strength, chemical resistance, and water repellency. However, excessive styrene content may increase the T_g_ of the polymer and reduce elasticity, which can negatively affect crack-bridging performance in waterproofing membranes. To balance these properties, styrene is often copolymerized with soft acrylic monomers such as butyl acrylate or ethyl acrylate, which lower the Tg and enhance flexibility and elongation. Hard acrylic monomers, including methyl methacrylate, may also be introduced to improve hardness and abrasion resistance when required [19].

Functional monomers such as acrylic acid, methacrylic acid, or hydroxyethyl acrylate are frequently added in small quantities to introduce polar functional groups that improve adhesion to mineral substrates and enable potential crosslinking reactions within the polymer network. These features can significantly enhance the membrane’s longevity and its ability to withstand environmental deterioration [20,21]. By selecting appropriate monomers and controlling the composition, A/S copolymers can be engineered to achieve the optimal balance between elasticity, mechanical strength, and environmental resistance required for high-performance waterproofing applications.

### 2.3. Particle Morphology (Core–Shell, Gradient, Single-Phase)

Particle morphology is another important factor influencing the performance of S/A latex dispersions. Modern emulsion polymerization techniques allow the production of polymer particles with various structural architectures, including single-phase particles, core–shell particles, and gradient morphologies. Core–shell latex particles consist of a soft polymer core surrounded by a harder outer shell. This structure enables a combination of flexibility and mechanical strength, making such systems particularly suitable for elastomeric waterproofing coatings. The soft core contributes to high elongation and crack-bridging capability, while the harder shell enhances resistance to abrasion and weathering [22].

**Figure 2:**
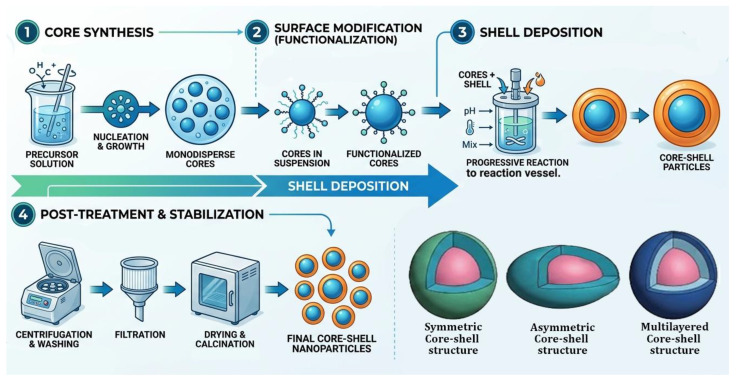
Schematic representation of core-shell structure general production process.

Gradient particle structures, in which the polymer composition gradually changes from the particle center to the surface, can further improve film formation and mechanical performance. Such advanced particle engineering approaches enable the development of high-performance coatings with optimized durability and environmental resistance (Figure 2) [23].

### 2.4. Influence of Tg and Polymer Composition on Membrane Flexibility

The T_g_ is a critical parameter controlling the mechanical behavior of elastomeric polymer membranes. The T_g_ represents the temperature at which a polymer transitions from a rigid glassy state to a flexible rubbery state. Polymers with lower T_g_ values typically exhibit higher flexibility and elongation, which are essential for crack-bridging applications in waterproofing membranes. Conversely, polymers with higher Tg values provide improved hardness and mechanical stability but may become brittle under low-temperature conditions [24]. In waterproofing applications, the T_g_ of S/A copolymers is precisely adjusted to keep the polymer in a rubbery state under normal operating temperatures. This balance between flexibility and strength is essential to maintain membrane integrity during thermal cycling and substrate movement [25].

However, for cold climate applications, the ability of the polymer to remain elastic at low temperatures and the actual temperature at which brittleness occurs (brittle point or zero ductility) are key design factors beyond Tg. Even a polymer that is flexible above Tg can lose its toughness and exhibit brittle failure at temperatures above the usual sub-zero operating limits. Quantitative evaluation through techniques such as dynamic mechanical analysis (DMA) at sub-ambient temperatures and direct measurement of the ductile–brittle transition provides a more accurate picture of mechanical behavior, guiding material selection and optimization of formulations for waterproofing membranes [26,27].

Functional monomers such as acrylic acid (AA), methacrylic acid (MAA), and hydroxy ethyl acrylate (HEA) play a critical role in determining the colloidal stability, viscosity, crosslinking potential, and adhesion of S/A dispersions. Even at low concentrations (0.5–5 wt%), these monomers introduce polar or reactive groups that significantly modify the polymer–particle and polymer–substrate interactions.

The monomers AA and MAA increase the surface charge density of latex particles, improving electrostatic stabilization and reducing coagulation during polymerization. Their presence also increases the viscosity of the dispersion due to swelling of the ionizable groups, particularly at alkaline pH. MAA, with its higher acidity and steric rigidity, enhances the potential for ionic or covalent crosslinking, contributing to improved mechanical strength and water resistance. The monomer HEA introduces hydroxyl groups capable of forming hydrogen bonds or participating in post-polymerization crosslinking reactions. This enhances adhesion to mineral substrates, improves film cohesion, and increases resistance to water swelling. However, excessive levels of functional monomers may increase water sensitivity or raise the minimum film-forming temperature (MFFT), requiring careful optimization [15,28].

### 2.5. Surfactants, Stabilizers, Plasticizers, and Rheology Modifiers

Besides monomer composition, the effectiveness of S/A emulsions is heavily affected by auxiliary formulation components, including surfactants, stabilizers, plasticizers, and rheology modifiers. Surfactants play a key role in stabilizing polymer particles during emulsion polymerization and preventing coagulation. However, excessive surfactant content may increase water sensitivity and reduce the durability of the final coating [24].

Plasticizers and coalescing agents are commonly added to reduce the MFFT of the polymer dispersion, allowing film formation under ambient conditions. Rheology modifiers such as Hydrophobically Modified Alkali-Swellable Emulsions (HASE) and hydrophobically modified ethoxylated urethanes (HEUR) are widely used to control viscosity and application properties in liquid-applied waterproofing membranes. These formulation components collectively determine the processing behavior, film formation, and long-term performance of elastomeric waterproofing systems [22,29].

Surfactants used in S/A emulsions are classified as anionic, cationic, nonionic, or amphoteric, with each type having a different effect on particle stability and film properties [30]. Typical concentrations range by weight of polymer solids [31]. To address problems such as surfactant migration, surface blooming, and increased water sensitivity, strategies such as the use of polymerizable (reactive) surfactants that are covalently bound to the polymer, the selection of surfactants with low water solubility, and careful control of the type and amount of surfactant during polymerization are applied. Such approaches contribute to maintaining durability, hydrophobicity, and long-term performance of the final coating [32].

## 3. Formulation of Water-Based Elastomeric Waterproofing Membranes

Water-based elastomeric waterproofing membranes are typically formulated as polymer-modified coating systems designed to form continuous, flexible, and impermeable films after drying. Their performance depends not only on the polymer binder but also on the careful combination of fillers, additives, rheology modifiers, and film-forming agents. The formulation strategy aims to achieve a balance between mechanical performance, crack-bridging capability, durability, and application properties.

In S/A systems, the binder dispersion acts as the primary structural component of the membrane, while fillers and additives tailor mechanical strength, viscosity, adhesion, and long-term stability. The interactions between these components determine the final film morphology and the waterproofing efficiency of the coating [13].

### 3.1. Typical Formulation Components

Water-based elastomeric waterproofing membranes generally consist of several key components, like polymer binder (S/A copolymer). Table 1 gathers the types of components that will be analyzed later on and can be used as binders, with their content (%) and their function. The polymer binder forms the continuous matrix of the membrane and largely determines its mechanical and barrier properties. S/A copolymers are widely used because they provide an excellent combination of flexibility, adhesion, and weather resistance. These binders typically exhibit T_g_ optimized for elastomeric behavior, enabling membranes to accommodate substrate movement and thermal cycling [14].

The binder content typically ranges between 30 and 60 wt% of the total formulation, depending on the desired elasticity and mechanical performance. Fillers and functional additives like mineral fillers play a crucial role in improving the mechanical stability and cost efficiency of waterproofing membranes. Common fillers include: CaCO_3_, silica, dolomite, lightweight fillers such as hollow microspheres.

Calcium carbonate is the most widely used filler due to its low cost and ability to enhance mechanical strength and dimensional stability. However, excessive filler loading may reduce elasticity and crack-bridging performance [15]. Rheology control is essential in liquid-applied membranes because the product must exhibit high viscosity during application while maintaining good leveling properties. Two major classes of rheology modifiers are widely used in water-based systems called HASE (Hydrophobically Modified Alkali-Swellable Emulsions) [33].

These polymers swell in alkaline conditions and provide viscosity control in aqueous formulations. HEUR (Hydrophobically Modified Ethoxylated Urethanes) thickeners operate through associative mechanisms, forming transient networks between polymer particles and improving sag resistance and application properties. Such rheology modifiers enable membranes to maintain a creamy consistency suitable for roller or brush application while preventing sedimentation of fillers [34]. During drying, polymer particles must coalesce to form a continuous film.

In some formulations, especially those containing high-Tg polymers, coalescing agents are added to facilitate this process. Coalescent reduces the MFFT of the dispersion, allowing film formation under ambient conditions. Typical coalescing agents include glycol ethers or ester-based solvents that temporarily plasticize the polymer particles. However, modern environmentally friendly formulations aim to minimize coalescent usage due to VOC restrictions, relying instead on optimized polymer design to achieve low MFFT values [17].

### 3.2. Development of Creamy-Viscosity Emulsion Systems

Liquid waterproofing membranes must exhibit rheological properties that allow easy application while preventing sagging or runoff on vertical surfaces. Achieving this balance requires careful control of particle interactions, filler dispersion, and thickener concentration. A typical formulation strategy involves the gradual addition of rheological modifiers during the mixing process to achieve a stable viscosity profile. The resulting system often exhibits pseudoplastic behavior, meaning that viscosity decreases under shear during application but recovers afterward. This shear-thinning behavior is essential for ensuring uniform film thickness and good substrate coverage [35].

**Figure 3:**
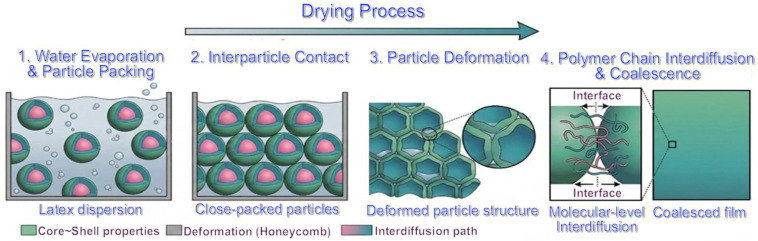
Schematic representation of the latex film formation mechanism, illustrating the particle packing (step 1), interparticle contact (step 2), particle deformation (step 3), and polymer chain inter diffusion (step 4) during drying process.

To complement the rheological behavior during application, the performance of liquid waterproofing membranes is also strongly influenced by the subsequent film-forming process during drying process. As illustrated in Figure 3, water evaporation (step 1) progressively brings the latex particles into close contact, leading to a densely packed structure (step 2). Continued drying (step 3) causes particle deformation, often leading to a polyhedral or honeycomb arrangement that enhances inter-particle contact. At this stage, the polymer chains start to inter-diffuse across the particle boundaries, reducing interfacial discontinuities (step 4). Eventually, complete fusion occurs, forming a continuous and uniform film with improved mechanical integrity and barrier performance. This transition from a fluid, shear-thinning system to a solid cohesive layer is essential to ensure the ultimate effectiveness of the membrane in protective applications.

### 3.3. Film Formation Mechanism and Coalescence

The waterproofing performance of S/A membranes is directly related to the film formation process that occurs during drying. Latex film formation typically occurs in three stages: (i) water evaporation—as water evaporates, polymer particles become closely packed; (ii) particle deformation—capillary forces cause particles to deform and fill interstitial voids; (iii) polymer interdiffusion—polymer chains diffuse across particle boundaries, forming a continuous film. This process results in a homogeneous elastomeric membrane capable of resisting water penetration and mechanical stress. Factors strongly influenced film formation, such as particle size, T_g_, coalescent content, and drying conditions [18].

### 3.4. Crack-Bridging Formulations—Strategies and Challenges

One of the most critical performance requirements for waterproofing membranes is crack-bridging capability, which refers to the ability of the membrane to maintain waterproofing integrity while spanning substrate cracks. Crack-bridging performance depends primarily on: polymer elasticity, elongation at break, film thickness, and adhesion to the substrate.

S/A membranes can typically achieve elongation values between 200 and 600%, enabling them to accommodate significant substrate movement without failure. However, excessive filler content or insufficient binder concentration can significantly reduce crack-bridging capacity. Modern formulations often incorporate optimized polymer dispersions with low Tg values and controlled particle morphology to enhance elasticity and durability. Depending on the conditions, reinforcing fibers or nano-fillers are also introduced to improve mechanical strength while maintaining flexibility.

## 4. Physico-Mechanical Properties of S/A Waterproofing Membranes

The physico-mechanical properties of water-based elastomeric waterproofing membranes determine their ability to provide long-term protection against water ingress while maintaining structural integrity under mechanical and environmental stresses. In S/A systems, these properties depend strongly on polymer composition, filler content, film morphology, and substrate interaction. Key performance parameters include tensile strength, elongation at break, crack-bridging capability, adhesion to substrates, water absorption, and thermal stability. These properties are essential for ensuring that the membrane can withstand substrate movement, thermal cycling, and environmental exposure without losing waterproofing performance [13].

### 4.1. Tensile Strength, Elongation at Break, and Elasticity

Tensile strength and elongation at break are among the most important mechanical properties for elastomeric waterproofing membranes. These parameters reflect the membrane’s ability to resist mechanical stress and accommodate deformation without failure. S/A membranes typically exhibit tensile strengths ranging between 1 and 5 MPa and elongation at break values between 200% and 600%, depending on polymer composition and filler content [36].

High elongation values are particularly desirable in waterproofing applications because they enable membranes to accommodate crack opening and substrate movement without rupture [37]. The elasticity of the membrane is primarily controlled by the T_g_ of the polymer binder. Lower Tg values lead to increased flexibility and improved crack-bridging performance, whereas higher Tg values increase stiffness and mechanical strength. Therefore, the polymer composition is typically optimized to achieve a balance between elasticity and structural stability [38]. In addition, the presence of mineral fillers can significantly influence mechanical properties. Moderate filler contents can improve tensile strength and dimensional stability, while excessive filler loading may reduce elasticity and increase brittleness [39,40]. Such properties can have an influence on waterproofing, as shown in Table 2, along with their typical range.

### 4.2. Water Absorption, Water Vapor Permeability, Capillary Penetration

Water resistance is a fundamental requirement for waterproofing membranes. The ability of a membrane to prevent water penetration depends on both the polymer matrix structure and the continuity of the formed film. S/A copolymers exhibit relatively low water absorption due to the hydrophobic nature of the styrene segments within the polymer chain. This hydrophobic character improves the barrier properties of the membrane and reduces capillary water transport [15]. Water vapor permeability is another important property, particularly in building envelope applications. While the membrane must prevent liquid water ingress, it should ideally allow controlled vapor diffusion to avoid moisture accumulation within the substrate. The water vapor transmission rate of acrylic membranes is influenced by factors such as polymer composition, film thickness, filler distribution, curing conditions. Properly designed S/A membranes can therefore provide an effective balance between water impermeability and vapor permeability, which is essential for maintaining long-term durability of building components.

### 4.3. Adhesion to Substrates (Concrete, Cement Mortars, Bitumen)

Reliable adhesion to construction substrates is critical for the long-lasting durability of waterproofing membranes. Poor adhesion may lead to delamination, blistering, and eventual failure of the waterproofing system. S/A dispersions exhibit excellent adhesion to a wide range of mineral substrates, including concrete, cement mortars, masonry, gypsum boards.

Adhesion mechanisms involve a combination of mechanical interlocking, surface wetting, and chemical interactions between functional groups in the polymer and the substrate surface. Functional monomers such as acrylic acid or hydroxy ethyl acrylate can significantly enhance adhesion by using polar groups interacting with hydroxyl groups present in cementitious substrates [37]. Surface preparation also plays a critical role in achieving optimal adhesion. Clean, dry, and properly primed substrates allow better penetration of the polymer dispersion into surface pores, improving mechanical anchoring and bonding strength.

### 4.4. Thermal Stability and Low-Temperature Flexibility

Waterproofing membranes must maintain their mechanical integrity under varying temperature conditions. Thermal cycling and seasonal temperature variations can cause expansion and contraction of building substrates, generating stresses within the waterproofing layer. Good thermal stability is typically demonstrated by S/A membranes due to the relatively stable polymer backbone and the presence of aromatic styrene units. However, their performance at low temperatures is primarily determined by the T_g_ of the polymer binder. Membranes with T_g_ values below service temperature remain flexible and capable of accommodating substrate movement. In contrast, membranes with higher Tg values may become brittle at low temperatures, leading to cracking and loss of waterproofing functionality [38]. Therefore, modern formulations are designed to maintain elasticity across a wide temperature range, ensuring reliable performance in both warm and cold climates [41].

### 4.5. Influence of Formulation Variables on Overall Performance

The physico-mechanical performance of elastomeric waterproofing membranes is strongly influenced by formulation variables such as binder content, filler loading, particle morphology and additive concentration. Higher polymer binder content generally improves elasticity, crack-bridging performance, and adhesion. Conversely, increasing filler concentration can improve mechanical strength but may reduce elongation and flexibility. Advanced polymer designs, including core–shell latex particles and self-crosslinking acrylic systems, have been developed to enhance mechanical performance while maintaining flexibility. These systems enable improved durability and resistance to environmental degradation in demanding construction applications [42,43].

## 5. Durability and Aging Behavior

The long-term durability of waterproofing membranes is a critical factor determining the reliability of building envelope protection systems. Water-based elastomeric membranes based on S/A copolymers are designed to withstand prolonged exposure to environmental stressors, including UV radiation, moisture, temperature fluctuations, and chemical pollutants. Durability depends on several factors such as polymer composition, cross-link density, filler content, and film morphology. Over time, environmental exposure may lead to physical and chemical changes within the polymer matrix, potentially affecting mechanical properties, adhesion, and waterproofing performance [44]. Understanding the aging mechanisms of S/A membranes is therefore essential for predicting service life and improving formulation strategies. Table 3 presents the impact on durability that membranes have when exposed to different factors, as well as the ways that they can be mitigated.

### 5.1. UV Resistance and Photodegradation of S/A Polymers

One of the primary factors responsible for the degradation of polymeric coatings exposed to outdoor environments is UV radiation. UV photons can break chemical bonds within the polymer backbone, initiating photooxidation reactions that lead to chain scission, crosslinking, and changes in mechanical properties. In S/A copolymers, the aromatic styrene units can absorb UV radiation, which can both protect and, under certain conditions, promote photodegradation. The actual photostability of these polymers therefore depends strongly on the specific formulation, including the type and number of stabilizers, as well as other additives that influence the polymer’s response to UV exposure [45]. However, prolonged UV exposure may still cause: Discoloration; Surface chalking; Embrittlement of the coating. 

**Figure 4:**
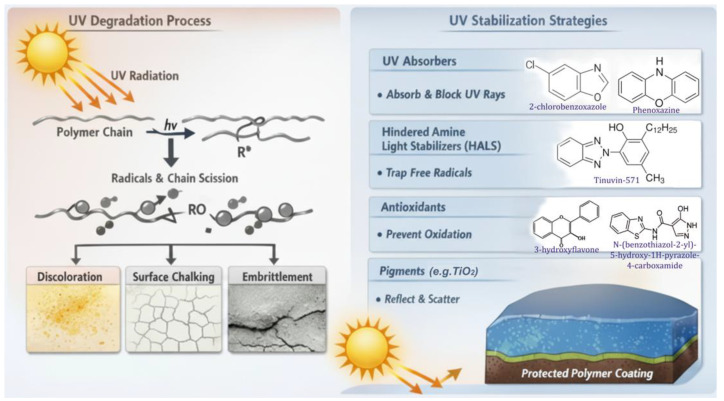
Schematic representation of the photo-oxidative degradation mechanisms and stabilization strategies in S/A polymer coatings.

Figure 4 indicates the photo-oxidative degradation mechanisms and stabilization strategies in S/A polymer coatings. Under UV irradiation (hν), polymer chains such as poly(styrene-co-acrylate)s undergo homolytic bond cleavage, forming alkyl radicals that react with atmospheric oxygen to generate peroxy radicals and hydroperoxides. These reactions lead to chain scission, oxidation, discoloration, surface chalking, and embrittlement. Stabilization is achieved through the incorporation of: (i) UV absorbers (e.g., benzotriazole derivatives) which attenuate harmful UV radiation; (ii) hindered amine light stabilizers (HALS, e.g., 2,2,6,6-tetramethylpiperidine derivatives) that act as radical scavengers; (iii) antioxidants (e.g., butylated hydroxytoluene, BHT) which inhibit oxidative degradation by decom-posing hydroperoxides; and (iv) inorganic pigments such as TiO_2_ that reflect and scatter UV radiation. The degra-dation mechanism is adapted from established literature on polymer photo-oxidation. 

To mitigate these effects, waterproofing formulations often include UV stabilizers, antioxidants, and light absorbers that slow down photochemical reactions within the polymer matrix [46]. The incorporation of pigments such as titanium dioxide can also enhance UV stability by reflecting and scattering UV radiation, thereby reducing degradation of the underlying polymer film.

### 5.2. Accelerated Weathering Protocols and Real-Life Exposure Data

To evaluate long-term durability within a reasonable timeframe, waterproofing membranes are commonly subjected to accelerated weathering tests. These tests simulate environmental conditions such as UV radiation, temperature variations, and moisture exposure. Common accelerated aging methods include QUV-accelerated weathering tests (such as UV exposure, condensation or water spray, temperature cycling), xenon arc exposure, and cyclic humidity and temperature testing. These tests enable researchers to track temporal changes in mechanical properties, colour stability, and waterproofing performance. Studies have shown that properly formulated acrylic membranes can maintain significant elasticity and waterproofing performance even after extended artificial weathering exposure [47]. Field exposure studies further confirm that elastomeric acrylic coatings can achieve service lifetimes exceeding 10–20 years in roofing and façade applications when applied in appropriate conditions.

### 5.3. Hydrolytic Stability and Long-Term Water Resistance

Continuous exposure to moisture and water immersion can influence the durability of polymeric waterproofing membranes. Hydrolytic degradation may occur when water molecules interact with susceptible chemical bonds within the polymer network. S/A copolymers generally demonstrate good hydrolytic stability due to the relatively stable carbon–carbon backbone of the polymer chain. However, water absorption may still lead to plasticization effects, which can temporarily reduce mechanical strength and increase permeability. The long-term water resistance of the membrane is strongly influenced by polymer hydrophobicity, crosslink density, film continuity, and filler distribution. Modern formulations often incorporate hydrophobic additives or optimized copolymer compositions to reduce water uptake and maintain dimensional stability under prolonged moisture exposure [37].

### 5.4. Crack-Bridging Durability over Cycles (Static and Dynamic)

A defining performance feature of elastomeric waterproofing membranes is their ability to maintain waterproofing performance while spanning cracks in the substrate. This property, known as crack-bridging capability, must remain effective throughout the service life of the membrane. Repeated thermal cycling, substrate movement, and environmental aging can gradually reduce the elasticity of the polymer film. Therefore, durability assessments often include cyclic crack-bridging tests in which the membrane is repeatedly stretched and relaxed while maintaining water impermeability. Research has shown that properly formulated S/A membranes can maintain crack-bridging performance over thousands of deformation cycles when sufficient polymer elasticity and film thickness are maintained [28].

### 5.5. Chemical Resistance (Alkalinity, Pollutants, CO_2_)

Environmental weathering is not the only factor that exposes waterproofing membranes to chemical agents; atmospheric pollutants, carbon dioxide, and alkaline substances from cementitious substrates also pose a threat. The chemical resistance of S/A membranes is largely determined by polymer composition and cross-link density. Acrylic polymers typically exhibit good resistance to alkaline environments, making them compatible with cement-based substrates commonly used in construction [48]. However, prolonged exposure to aggressive chemicals may still lead to gradual degradation of the polymer matrix. As a result, formulation strategies often include stabilizing additives and protective pigments to improve chemical durability.

## 6. Comparison with Alternative Waterproofing Technologies

**Figure 5:**
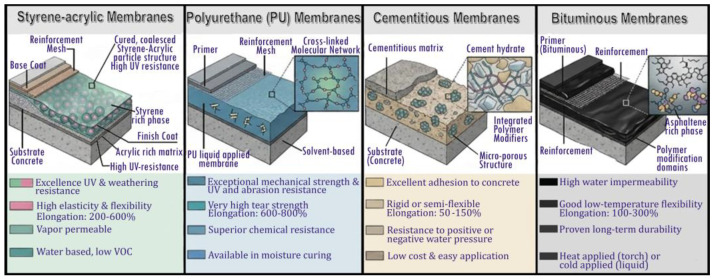
Comparative analysis of major liquid-applied waterproofing technologies, including S/A, PU, cementitious, and bituminous membranes, together with schematic representations of the corresponding composite systems [30,49,50,51,52,53].

Liquid-applied waterproofing membranes encompass a broad range of technologies developed to protect building structures against water pressure. Among the most widely used systems are S/A elastomeric coatings, PU membranes, cementitious waterproofing slurries, and bituminous emulsions. Each of these technologies presents distinct advantages and limitations in terms of mechanical performance, durability, cost, and environmental impact. S/A waterproofing membranes have gained significant market share due to their favorable balance between performance, ease of application, and environmental compatibility. However, alternative technologies remain widely used in specific construction applications depending on the required mechanical properties and exposure [49] (Figure 5). As depicted in Table 4, there are different technologies that can help with the elongation, UV resistance, and the minimization of VOCs. Moreover, Table 4 demonstrates the typical use of these technologies as far as the materials are concerned.

### 6.1. Polyurethane Liquid Membranes

PU-based liquid membranes are widely used in roofing, balconies, and exposed waterproofing systems due to their excellent mechanical strength, high elasticity, and strong adhesion to a variety of substrates. PU membranes typically exhibit elongation values exceeding 600–800%, which allows them to accommodate significant substrate movement without cracking. In addition, they offer superior abrasion resistance and chemical stability compared with many acrylic-based systems [50,51,52]. 

However, PU systems also present several limitations. Many PU formulations contain solvent-based components or reactive isocyanates, which may raise environmental and health concerns during application. Furthermore, PU membranes generally require stricter surface preparation and controlled curing conditions compared with water-based acrylic systems [48]. Consequently, while PU membranes provide excellent mechanical performance, water-based S/A systems are often preferred in applications where low VOC emissions, ease of application, and environmental compliance are priorities.

### 6.2. Cementitious Waterproofing Slurries (1K and 2K)

Cementitious waterproofing systems are widely used in construction due to their compatibility with concrete substrates and their ability to withstand hydrostatic pressure. These systems typically consist of cement-based powders combined with polymer modifiers such as acrylic or styrene–butadiene latex. Two main types of cementitious membranes are commonly used: single-component (1K) cementitious coatings and two-component (2K) polymer-modified cementitious membranes [53,54,55].

Cementitious systems provide excellent adhesion to mineral substrates and good resistance to water pressure. However, their limited elasticity and crack-bridging capacity can restrict their performance in applications where significant substrate movement occurs. Polymer-modified cementitious membranes have been developed to improve flexibility and durability, but they still generally exhibit lower elongation values compared with elastomeric acrylic or PU membranes [49].

### 6.3. Bituminous Emulsions

Bituminous waterproofing membranes have historically been among the most widely used waterproofing solutions in construction. These systems are based on modified asphalt binders that provide strong water resistance and durability. Bituminous systems are typically available in two main forms: sheet membranes, bituminous emulsions, or liquid coatings. Bitumen-based membranes offer excellent waterproofing performance and good resistance to water penetration. However, they often require heat applications or torching during installation, which increases safety risks and limits their suitability for certain applications. Furthermore, bituminous materials can exhibit reduced flexibility at low temperatures and may degrade under prolonged UV exposure unless protected by additional coatings or protective layers [49].

### 6.4. Hybrid Polymer Systems (S/A–PU Hybrids, Silane-Modified Polymers)

Recent advances in waterproofing technology have led to the development of hybrid polymer systems that combine the advantages of different polymer chemistries. Examples include: S/A/PU hybrid coatings, silane-modified polymer (SMP) membranes, acrylic–silicone hybrid coatings. These hybrid systems aim to combine the elasticity of PU, the weather resistance of acrylic polymers, and the adhesion properties of silane-modified polymers. As a result, they can offer improved mechanical performance and enhanced durability in demanding construction environments. However, hybrid formulations are typically more complex and expensive than conventional acrylic systems, which may limit their widespread use in cost-sensitive applications [48].

### 6.5. Cost–Performance–Sustainability Matrix

The selection of a waterproofing system ultimately depends on a combination of performance requirements, environmental considerations, and economic factors. Water-based S/A membranes are widely regarded as a cost-effective and environmentally friendly solution for many waterproofing applications. Their advantages include: low VOC emissions, easy application with standard tools, good elasticity and crack-bridging capability, compatibility with cementitious substrates.

In contrast, PU systems typically offer superior mechanical performance but at higher material cost and with stricter application requirements. Cementitious membranes provide excellent compatibility with mineral substrates but are generally less flexible. Bituminous membranes remain attractive due to their proven durability and relatively low cost, but may present environmental and installation challenges. Accordingly, selecting the ideal waterproofing solution requires careful evaluation of its performance, environmental impact, and total lifecycle cost [56,57,58].

## 7. Advances in the Field

Continuous developments in polymer chemistry and coating technology have led to significant improvements in the performance of water-based elastomeric waterproofing membranes. Research efforts focus on enhancing mechanical durability, environmental compatibility, and multifunctional performance through advanced polymer architectures, nanomaterials, and innovative additives. Recent advances in S/A systems include self-crosslinking polymer dispersions, nano-reinforced coatings, silicone-modified binders, and smart functional additives designed to improve barrier properties, crack-bridging capability, and long-term environmental stability [59]. 

### 7.1. Next-Generation Polymer Binders (Self-Crosslinking, Nano-Reinforced, Silicone-Modified)

One of the most significant developments in acrylic coating technology is the design of self-crosslinking polymer systems. These systems incorporate reactive functional groups that form cross-linked polymer networks during film formation, resulting in improved mechanical strength and chemical resistance. Self-crosslinking acrylic dispersions can significantly enhance abrasion resistance, water resistance, mechanical stability, and solvent resistance. Another important development is the design of core–shell latex particles, where the inner core provides elasticity while the outer shell improves durability and weather resistance. Such advanced particle architectures allow better control of mechanical performance and film formation in elastomeric membranes [37].

### 7.2. Nanomaterials for Improved Mechanical Strength and Barrier Properties

Nanotechnology has emerged as a promising approach for improving the performance of polymer coatings. The incorporation of nanomaterials into S/A membranes can enhance mechanical strength, barrier properties, and durability. Common nanomaterials investigated for waterproofing coatings include: nano-silica (SiO_2_), nano-clay, carbon nanotubes, graphene derivatives. Nano-silica particles, for example, can improve tensile strength and reduce permeability by increasing the tortuosity of diffusion pathways within the polymer matrix. Similarly, layered nano-clays can enhance barrier properties and improve resistance to water penetration and chemical attack [28]. However, achieving uniform dispersion of nanoparticles within the polymer matrix remains a key challenge in the development of nano-reinforced coatings [60].

One of the main problems in nano-reinforced coatings is the tendency of nanoparticles to form aggregates in aqueous solutions. Such aggregates can cause defects in the film, such as voids, lumps, or uneven surfaces, reducing both mechanical strength and barrier effectiveness. The application of appropriate methods to control the dispersion of nanoparticles, like surface modification, the use of disintegrants, or the optimization of mixing techniques, is essential to create uniform films and fully exploit the advantages of nanofillers in polymer coatings [61,62].

### 7.3. Low-VOC and VOC-Free Systems Compliant with Latest Environmental Standards

Environmental regulations and sustainability initiatives have driven the development of low-VOC and VOC-free waterproofing technologies. Traditional solvent-based coatings are increasingly being replaced by water-based polymer dispersions that provide comparable performance with significantly reduced environmental impact. Recent research focuses on reducing or eliminating coalescing solvents traditionally used to assist film formation in latex systems [63,64]. Advances in polymer design allow the production of dispersions with lower MFFT, enabling film formation under ambient conditions without the need for high levels of coalescing agents [38]. Such developments align with green building certification systems such as LEED and BREEAM, which promote the use of environmentally friendly construction materials.

### 7.4. Enhancements in Crack-Bridging and Long-Term Elasticity Technologies

Enhancing crack-bridging capability remains a key research objective for elastomeric waterproofing membranes. Recent advances focus on optimizing polymer composition and particle morphology to achieve improved flexibility and fatigue resistance. Strategies for improving crack-bridging performance include: low-Tg polymer formulations, optimized binder-to-filler ratios, and incorporation of reinforcing fibers or micro-fillers. These approaches aim to maintain high elongation and elasticity even after prolonged environmental aging, ensuring reliable waterproofing performance under cyclic mechanical loading [38].

### 7.5. Smart or Functional Additives (Hydrophobicity Boosters, Anti-Mold Agents)

**Figure 6:**
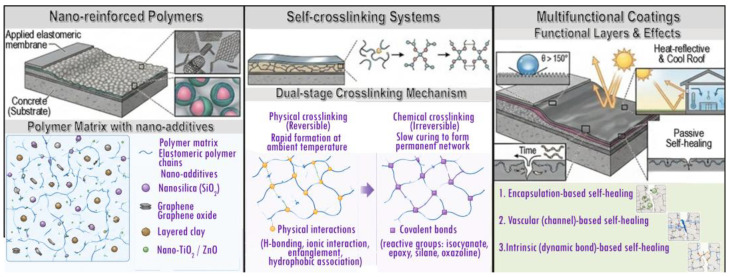
Emerging technologies in elastomeric waterproofing coatings include nano-reinforced polymers, self-crosslinking systems, and multifunctional coatings.

Another emerging area in waterproofing technology is the development of functional coatings with additional protective properties. Researchers are increasingly incorporating additives that provide functionalities beyond traditional waterproofing. Examples include: hydrophobic agents that enhance water repellency, anti-microbial additives that prevent biological growth, self-cleaning photocatalytic particles such as TiO_2_, and infrared-reflective pigments that improve thermal performance of roofing coatings. Such multifunctional coatings can contribute to improved durability and energy efficiency of buildings while maintaining the fundamental waterproofing function of the membrane (Figure 6, Table 5).

## 8. Applications and Case Studies

Water-based elastomeric waterproofing membranes based on S/A copolymers are widely used in modern construction due to their versatility, environmental compatibility, and ease of application. These systems are typically applied as liquid coatings that cure to form seamless waterproof barriers, providing protection against water infiltration across a wide range of building components. Their ability to conform to complex geometries, bridge cracks, and adhere strongly to mineral substrates makes them suitable for both new construction and refurbishment projects. As a result, S/A waterproofing membranes are commonly used in applications such as roofing systems, wet rooms, foundations, and façade protection [37].

Table 6 summarizes a set of key research directions in which S/A membranes are applied, as well as their corresponding functional performance objectives. Nano-reinforced membranes mainly aim to improve barrier properties, increasing resistance to gas and moisture penetration. Self-healing coatings focus on damage repair, enabling the material to heal microcracks and extend its lifespan. At the same time, biopolymers are studied with sustainability in mind, seeking to limit environmental burdens through the use of renewable raw materials. Finally, smart coatings are developed to offer multifunctional protection, incorporating adaptive or responsive properties that enhance their overall performance in different operating environments. Table 7 summarizes the main research directions for S/A membranes and their respective goals. It includes applications such as nano-reinforced membranes for barrier enhancement, self-healing coatings for damage repair, biopolymers with an emphasis on sustainability, and smart coatings for multifunctional protection.

### 8.1. Roof Waterproofing

Roof waterproofing represents one of the most important applications of elastomeric liquid membranes. Flat and low-slope roofs are particularly vulnerable to water infiltration due to standing water, thermal expansion, and environmental expansion. S/A membranes provide several advantages in roof applications, including: seamless waterproofing without joints, excellent crack-bridging capability, high resistance to UV radiation and weathering, and compatibility with various roofing substrates. These membranes are typically applied by roller, brush, or spray to form a continuous coating layer that prevents water penetration while maintaining flexibility during thermal cycling. In addition, reflective pigments may be incorporated to improve solar reflectance and reduce heat absorption on exposed roof surfaces [48].

### 8.2. Wet Rooms and Interior Waterproofing

Interior waterproofing applications such as bathrooms, kitchens, and laundry areas require membranes that provide reliable protection against moisture while remaining compatible with ceramic tile systems. S/A waterproofing coatings are widely used beneath ceramic tiles due to their strong adhesion to cementitious substrates and compatibility with tile adhesives. These systems are typically applied as thin liquid membranes that are cured to form flexible waterproof layers beneath tile coverings.

Standards such as EN 14891 specify performance requirements for liquid-applied waterproofing products used under ceramic tiling systems, including tests for crack bridging, adhesion, and water impermeability. The use of water-based acrylic membranes in wet rooms has increased significantly due to their low VOC content, easy application, and rapid drying characteristics [49].

### 8.3. Foundations & Retaining Walls

Waterproofing of below-ground structures is essential to prevent water infiltration and protect building foundations from moisture-related damage. S/A membranes can be applied to foundation walls and retaining structures to form protective waterproof barriers. In these applications, the membrane must resist hydrostatic pressure, soil moisture, and chemical exposure from surrounding soils. Although cementitious and bituminous systems are traditionally used in foundation waterproofing, elastomeric acrylic coatings offer advantages in terms of flexibility and ease of application. Their ability to accommodate minor substrate movement helps reduce the risk of cracking and leakage in foundation systems [49].

### 8.4. Compatibility with Cementitious Overlays, Screeds, and Insulation Systems

Another important application area for S/A waterproofing membranes is their use in combination with cementitious screeds, mortars, and thermal insulation systems. These membranes are often incorporated into multilayer building envelope systems, where they act as intermediate waterproofing layers beneath protective coatings or finishing materials. Their compatibility with cement-based materials allows them to be integrated with: polymer-modified mortars, insulation boards, reinforced cementitious coatings. Such multilayer systems are commonly used in façade protection and terrace waterproofing applications, where both structural protection and aesthetic finishes are required [37].

### 8.5. Practical Considerations During Application (Substrate Prep, Humidity, Curing)

The performance of liquid-applied waterproofing membranes depends not only on formulation but also on proper application procedures. Key factors affecting membrane performance include: substrate preparation, ambient temperature and humidity, and film thickness and curing conditions. Proper substrate preparation is essential to ensure strong adhesion. Surfaces should be clean, dry, and free of contaminants such as dust, oils, or loose particles. In some cases, primers are applied prior to the membrane to improve adhesion and substrate penetration. Additionally, the membrane must be applied at sufficient thickness to achieve the required waterproofing performance. Insufficient coating thickness may result in pinholes or discontinuities that compromise the integrity of the waterproofing layer [65].

## 9. Challenges, Limitations, and Future Research Directions

Despite significant advances in water-based S/A elastomeric waterproofing membranes, challenges remain regarding long-term durability, environmental sustainability, and performance under harsh service conditions. Future research is expected to focus on improved polymer design, enhanced resistance to environmental stressors, and more sustainable formulations. Increasing demand for durable and eco-friendly construction materials continues to drive innovation in advanced polymer-based waterproofing systems with improved mechanical performance and extended service life [15].

One of the main challenges associated with elastomeric waterproofing membranes is maintaining long-term performance under severe environmental conditions. Exposure to UV radiation, temperature fluctuations, moisture, and atmospheric pollutants may gradually degrade polymeric coatings, leading to loss of elasticity, reduced adhesion, and increased permeability [66,67]. Although S/A systems generally exhibit good resistance to weathering, prolonged environmental exposure can still induce polymer degradation through photooxidation and hydrolytic processes. Future research should therefore focus on improving polymer stability through optimized copolymer compositions and the incorporation of stabilizing additives such as UV absorbers and antioxidants [48]. Another promising research direction involves the development of self-healing polymer coatings capable of restoring their structural integrity after mechanical damage or crack formation [68]. 

Enhancing UV stability while maintaining high elasticity remains a significant formulation challenge. Increasing the styrene content in acrylic copolymers may improve UV resistance but often lead to increased stiffness and reduced elongation [69]. Future research should focus on designing polymer architectures that provide both photochemical stability and elastomeric flexibility. Strategies may include the development of hybrid copolymers incorporating silicone or fluorinated segments that enhance UV resistance without compromising mechanical performance [37]. The use of advanced UV stabilizers and nano-scale UV absorbers may also contribute to improved long-term weathering resistance in elastomeric coatings. 

**Figure 7:**
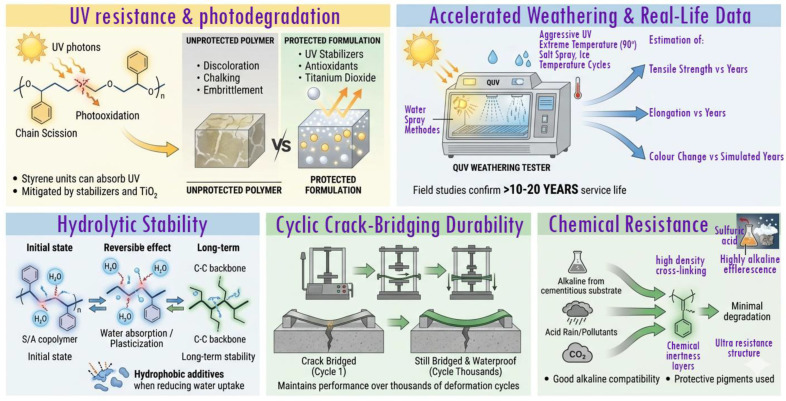
Schematic representation of tests and results related to enhancing long-term durability of polymer waterproofing membranes [45,46,47,48,66,67,68,69,70,71,72,73,74].

Figure 7 illustrates a schematic representation of tests and results related to enhancing long-term durability of polymer waterproofing membranes. Polymer waterproofing membranes such as PVC, ethylene-vinyl-acetate (EVA), TPO and polyolefin elastomers (POE), EPDM, PU, and polymer-modified bitumen systems) degrade over long-term exposure to extreme environmental conditions through a combination of UV radiation, moisture, temperature fluctuations, mechanical loading, and chemical exposure. The literature consistently shows that durability is governed less by the base polymer alone and more by formulation additives, multi-stress interactions, and interface stability. Under UV exposure, degradation is driven by photo-oxidation, chain scission, and additive depletion. PVC is particularly sensitive due to dehydrochlorination and plasticizer loss, while TPO/POE systems exhibit improved but not complete resistance, and EPDM can still degrade due to impurity-initiated oxidation. UV resistance is therefore strongly formulation-dependent, especially on stabilizers such as HALS and UV absorbers. Accelerated weathering tests (UV, heat, and humidity) are widely used but only partially replicate field performance. Real-life exposure introduces nonlinear and synergistic effects, where combined stresses produce more severe degradation than single-factor laboratory tests. Field studies show that mechanical property loss is often underestimated in simplified accelerated aging protocols [69,70,71,72].

Hydrolytic stability varies significantly across polymers. PU is most vulnerable due to hydrolysis of urethane bonds, especially under warm and humid conditions. PVC is affected mainly through plasticizer leaching and interface weakening, while TPO and POE show comparatively better resistance. Moisture also accelerates oxidative degradation by enhancing diffusion and radical mobility. Cyclic crack-bridging durability is controlled by fatigue mechanisms under repeated mechanical loading combined with environmental aging. UV-pre-damage significantly reduces ductility and accelerates crack propagation. Static tensile properties are insufficient predictors of long-term performance, as fatigue and microcrack accumulation govern failure in real conditions. Chemical resistance depends on the specific exposure environment. EPDM performs well against acids and ozone but is more sensitive to UV degradation. PVC is vulnerable to hydrocarbon-induced plasticizer extraction, while TPO/POE generally offer the best-balanced resistance. Polyurethane systems are particularly sensitive to hydrolysis and certain solvents [72,73,74].

Sustainability considerations are increasingly influencing the development of construction materials. Conventional polymer dispersions are typically produced from petroleum-based monomers, raising concerns regarding environmental impact and resource depletion. Future research directions include the development of bio-based acrylic monomers and renewable polymer dispersions derived from sustainable feedstocks. Such materials could reduce the carbon footprint of waterproofing systems while maintaining comparable performance characteristics [38]. Additionally, research into solvent-free and low-energy polymerization processes may contribute to more sustainable manufacturing of latex dispersions used in waterproofing applications.

Although several standards are available for evaluating waterproofing membranes, differences in testing methods and environmental conditions often hinder direct performance comparisons. Standards such as EN 14891 and EAD 030352-00-0503 specify requirements for liquid-applied waterproofing systems beneath ceramic tiles. However, improved testing protocols are still needed to better simulate long-term environmental exposure and mechanical stress. Future studies should therefore emphasize advanced aging and fatigue testing to more accurately predict the real-life performance of elastomeric waterproofing membranes.

Future developments in S/A waterproofing membranes are expected to focus on multifunctional coatings with enhanced durability and protective functions. Emerging technologies include nano-reinforced polymers, self-healing elastomeric membranes, smart sensing coatings, and energy-efficient reflective membranes. These innovations aim to improve sustainability, durability, and overall performance of modern building envelopes. Continued collaboration among polymer scientists, materials engineers, and construction professionals will be essential for advancing next-generation elastomeric waterproofing systems.

## 10. Conclusions

Water-based elastomeric waterproofing membranes based on S/A copolymers constitute a versatile and increasingly relevant class of construction materials, widely applied in roofing, wet areas, facades, and foundation protection due to their favorable balance of environmental compatibility, flexibility, adhesion, and ease of application.

This review focused on key performance-determining systems, highlighting that the main governing factors are polymer chemistry, formulation design, particle morphology, binder-to-filler ratio, and film formation behavior. Overall, system performance is primarily reflected in enhanced crack-bridging ability, water resistance, and long-term durability, provided that these parameters are appropriately optimized. Compared with PU-, cementitious-, and bitumen-based systems, S/A membranes consistently show a more balanced performance profile in terms of cost, sustainability, and application safety, largely due to their water-based and low-VOC formulation.

From a technological perspective, the most significant advances arise from self-crosslinking dispersions, nano-reinforcement strategies, and multifunctional additive systems, which collectively improve mechanical strength and durability performance. In parallel, sustainability-driven developments based on renewable raw materials and greener production routes further extend the applicability of these systems within modern environmental frameworks.

Future perspectives indicate that the main research direction will focus on improving long-term durability under extreme environmental exposure, refining predictive testing methodologies for service life assessment, and advancing material design through nano-engineered and smart multifunctional coatings. Continued interdisciplinary collaboration will be essential to translate these developments into next-generation waterproofing systems with enhanced durability, sustainability, and functional performance.

## 11. Literature Review Methodology

A systematic literature search was carried out to ensure a complete and objective review. Relevant publications were identified through international scientific databases, such as Scopus, Web of Science, and Google Scholar, using keyword combinations such as “wet-applied membranes”, “waterproofing coatings”, “acrylic latex”, “PU coatings”, and “emulsion polymerization”. The search focused mainly on the period 2000–2025, while older fundamental studies were also included, where necessary. After removing duplicate records, a title and abstract check was performed, and then the full text was evaluated.

The selection of studies was based on their direct relevance to wet-applied membranes and related polymeric coating systems, with an emphasis on experimental and review works examining material properties, performance, and applications. Studies with no direct relevance to the subject (such as unrelated nanomaterials or biomedical polymers) were excluded. To limit potential bias, recent and frequently cited publications in peer-reviewed scientific journals were preferred, while the bibliographic references of key articles were also examined to identify additional relevant sources.

## Figures and Tables

**Table 1:** Typical monomer composition ranges (wt%) in S/A copolymers and corresponding T_g_.

Styrene (wt%)	Acrylic Monomer Type (wt%)	Typical Acrylic Examples	Approx. Tg (°C)	Applications
20–30%	70–80% soft acrylic	Butyl acrylate (BA), 2-ethylhexyl acrylate (2-EHA)	−20 to 0	Very flexible, pressure-sensitive adhesives
30–40%	60–70% soft acrylic	BA, EHA	0 to 20	Flexible coatings, sealants
40–50%	50–60% mixed acrylic	BA + methyl methacrylate (MMA)	10 to 40	Balanced flexibility/hardness, general-purpose binders
50–60%	40–50% mixed acrylic	MMA + BA	30 to 60	Architectural coatings, good durability
60–70%	30–40% hard acrylic	MMA, ethyl acrylate (EA)	50 to 80	Hard coatings, improved block resistance
70–80%	20–30% hard acrylic	MMA-rich	70 to 100	High hardness, industrial coatings
80–90%	10–20% hard acrylic	Mostly styrene + MMA	90 to 110	Very hard, brittle systems
0–10%	90–100% soft acrylic	BA, EHA	−50 to −20	Extremely soft, tacky materials

**Table 2:** Comparison between the binder’s components, their function, and their typical content (%).

Component	Function	Typical Content (%)
S/A binder	Film formation	30–60
CaCO_3_ filler	Mechanical stability	20–40
Rheology modifier (HASE/HEUR)	Viscosity control	0.3–1
Coalescent	Film formation	1–5
Additives	Stabilization	<2

**Table 3:** The influence on waterproofing according to the property, with the typical range, collectively.

Property	Typical Range	Influence on Waterproofing
Tensile strength	1–5 MPa	Mechanical resistance
Elongation	200–600%	Crack bridging
Water absorption	<10%	Impermeability
Adhesion to concrete	>1 MPa	Durability

**Table 4:** This is a summary table on the effects that different exposure factors can have on the membranes, and their mitigation strategies.

Exposure Factor	Effect on Membrane	Mitigation Strategy
UV radiation	Chain scission	UV stabilizers
Moisture	Plasticization	Hydrophobic additives
Thermal cycling	Fatigue	Low T_g_ polymers
Pollutants	Oxidation	Antioxidants

**Table 5:** Summary of emerging technologies in elastomeric waterproofing coatings and their applications, highlighting the key performance requirements and the advantages provided by self-adhesive (S/A) membrane systems.

Emerging Technology	Application	Key Requirement	Advantages of S/A Membranes and Elastomeric Waterproofing Coatings
Nano-reinforced polymers	Roofs	UV resistance	Enhanced elastomeric behavior, improved mechanical strength, higher UV durability, and longer service life
Self-crosslinking systems	Wet rooms	Adhesion to tiles	Improved compatibility with mortars, stronger adhesion, seamless curing, and better chemical/moisture resistance
Multifunctional coatings	Foundations	Water pressure	Flexible barrier with combined waterproofing, crack-bridging, corrosion resistance, and durability under hydrostatic pressure
Multifunctional coatings	Façades	Weather resistance	Breathable coating with enhanced weatherability, self-cleaning potential, thermal regulation, and resistance to environmental aging

**Table 6:** Overview of advanced coating technologies, summarizing their key benefits and main implementation challenges.

Technology	Benefit	Challenge
Nano-silica reinforced coatings	Improved barrier properties	Dispersion issues
Self-crosslinking acrylics	Higher durability	Higher cost
Silicone-modified acrylics	Improved hydrophobicity	Formulation complexity
Smart coatings	Multifunctional protection	Early research stage

**Table 7:** Summary of typical research areas where S/A membranes can be used, and their performance purpose.

Research Area	Objective
Nano-reinforced membranes	Improved barrier properties
Self-healing coatings	Damage recovery
Bio-based polymers	Sustainability
Smart coatings	Multifunctional protection

## Data Availability

Data sharing is not applicable to this article as no new data were created or analyzed in this study.

## Abbreviations

The following abbreviations are used in this manuscript:

1K	One-Component System
2K	Two-Component System
BHT	Butylated hydroxytoluene
BREEAM	Building Research Establishment Environmental Assessment Method
DIN EN 14891	European Standard for Waterproofing Under Tiles
EAD 030352-00-0503	European Assessment Document
EN 14891	European Norm 14891
EPDM	Ethylene Propylene Diene Monomer
ETAG 022	European Technical Approval Guideline
EVA	Ethylene-vinyl-acetate
HALS	Hindered amine light stabilizers
HASE	Hydrophobically Modified Alkali-Swellable Emulsion
HEUR	Hydrophobically Modified Ethoxylated Urethane
LAMs	Liquid-Applied Membrane(s)
LEED	Leadership in Energy and Environmental Design
MFFT	Minimum Film-Forming Temperature
POE	Polyolefin elastomers
PU	Polyurethane
PVC	Polyvinyl Chloride
S/A	Styrene–Acrylic
SBR	Styrene–Butadiene Rubber
SMP	Silane-Modified Polymer
T_g_	Glass Transition Temperature
TPO	Thermoplastic Polyolefin
UV	Ultraviolet
VOC	Volatile Organic Compounds
